# Many-Body Models for Chirality-Induced Spin Selectivity in Electron Transfer

**DOI:** 10.1021/acs.nanolett.4c02912

**Published:** 2024-09-22

**Authors:** Alessandro Chiesa, Elena Garlatti, Matteo Mezzadri, Leonardo Celada, Roberta Sessoli, Michael R. Wasielewski, Robert Bittl, Paolo Santini, Stefano Carretta

**Affiliations:** † Dipartimento di Scienze Matematiche, Fisiche e Informatiche, Università di Parma, I-43124 Parma, Italy; ‡ INFN−Sezione di Milano-Bicocca, gruppo collegato di Parma, 43124 Parma, Italy; ¶ Consorzio Interuniversitario Nazionale per la Scienza e Tecnologia dei Materiali (INSTM), I-50121 Firenze, Italy; § Dipartimento di Chimica “U. Schiff” (DICUS), Università degli Studi di Firenze, I-50019 Sesto Fiorentino (FI), Italy; ∥ Department of Chemistry, Center for Molecular Quantum Transduction, and Institute for Sustainability and Energy at Northwestern, Northwestern University, Evanston, Illinois 60208-3113, United States; ⊥ Fachbereich Physik, Berlin Joint EPR Lab, Freie Universität Berlin, D-14195 Berlin, Germany

**Keywords:** Chirality-Induced Spin-Selectivity, Electron Transfer, Spin Polarization, Many-Body Models, Electron Correlations

## Abstract

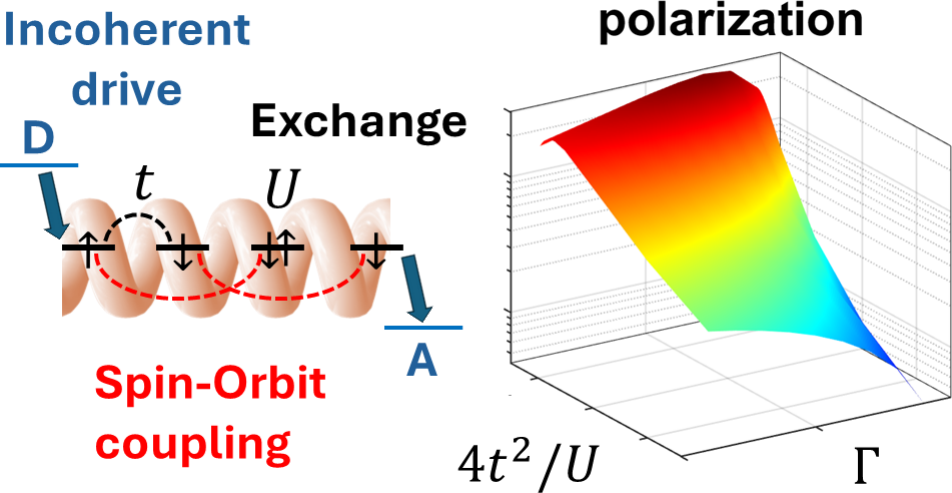

We present the first microscopic model for the chirality-induced spin selectivity effect in electron-transfer, in which the internal degrees of freedom of the chiral bridge are explicitly included. By exactly solving this model on short chiral chains we demonstrate that a sizable spin polarization on the acceptor arises from the interplay of coherent and incoherent dynamics, with strong electron–electron correlations yielding many-body states on the bridge as crucial ingredients. Moreover, we include the coherent and incoherent dynamics induced by interactions with vibrational modes and show that they can play an important role in determining the long-time polarized state probed in experiments.

The Chirality-Induced Spin Selectivity (CISS) effect is a stunning but still not understood phenomenon in which electrons are strongly spin polarized when passing through chiral molecules. It attracted an increasing interest^1,2^ for explaining chemical reactions^3^ or biological processes^1,4^ and for applications in spintronics^5^ and quantum technologies.^6,7^

CISS was detected on a variety of systems,^1^ using different experimental techniques.^1,8,9^ Many models were proposed to interpret transport and photoemission experiments,^10^ but a univocal explanation is still lacking.^11^ Spin polarization is originated by spin–orbit coupling (SOC),^12−14^ typically small in organic systems. Hence, additional interactions are needed to amplify spin polarization to the observed level. Single-electron models^12,13,15−20^ generally yield small spin polarization with strong decoherence. Models for transport indicate that this can be enhanced^10,21−26^ by electron–electron^21,23^ or electron–vibration^22,27−29^ interactions.

To disentangle the role of various ingredients, a different approach exploiting a simpler setup was recently proposed.^30−32^ Photoinduced electron transfer (ET) through a chiral molecular bridge enables to remove complex interfaces or metals with large SOC and to focus on the bridge. Fay and Limmer theoretically showed that an exchange interaction between the moving electron and the unpaired one on the donor can lead to a spin polarization.^33^ However, such interaction is lacking in transport or photoemission experiments. Moreover, this model focuses on the initial and charge-separated states, without a microscopic description of the internal degrees of freedom of the bridge. Very recently, CISS was observed in photoinduced ET.^34^ This finding definitely moves the focus to the only player present in all experiments, i.e. the chiral bridge, whose explicit description in ET is still lacking.^31,33^

Here we provide such a microscopic description by a Fermionic many-body model explicitly accounting for the internal degrees of freedom of the bridge. The large spin polarization (above 50%) observed in ET^34^ and transport measurements^35^ on short chiral molecules indicates that the underlying mechanism should be captured by a minimal model on a limited number of sites, which therefore is our focus. In contrast with transport models,^21,29^ this permits an exact numerical solution of the ET dynamics, including electron–electron interactions, coherent and incoherent coupling with vibrations. Such a minimal model provides a deep insight into the spin polarization mechanism and allows us to explore different regimes of parameters.

We obtain a sizable spin polarization with reasonable parameters and highlight the crucial role of electron–electron interactions, consistently with proposals for transport.^10,21,22^ Finally, we investigate the long-time relaxation of the donor-chiral bridge-acceptor supramolecule, establishing opposite local spin polarization on donor and acceptor with the bridge in the ground singlet, as experimentally observed.^34^

## Model System

**Figure 1:**
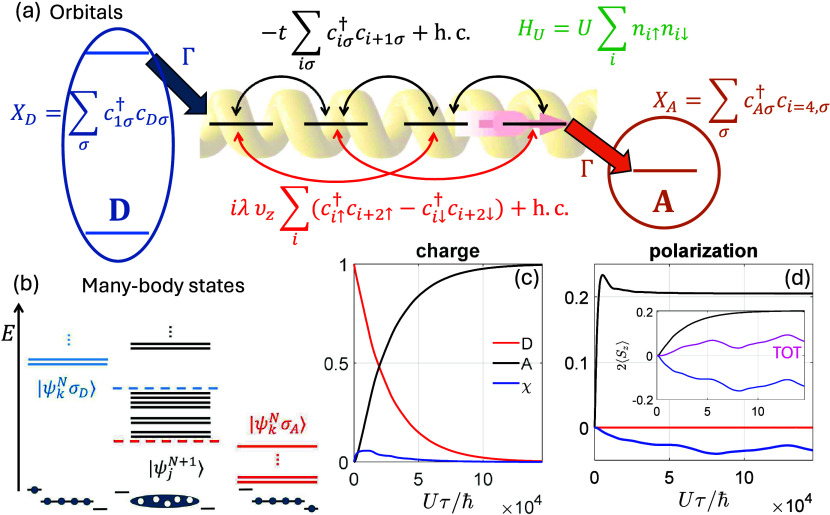
**System description and spin polarization dynamics**. (a) Model system, consisting of a 4-site chiral bridge with nearest-neighbor spin-independent hopping (black), next-to-nearest-neighbor SOC (red, only the most relevant *z* component included) and on-site Coulomb repulsion (green). Sites 1 and 4 are coupled to the donor (excited) and acceptor states by an incoherent spin-independent hopping driven by a large energy gap, characterized by a rate Γ. (b) Energy level structure of the bridge many-body states with *N* + 1 (black) electrons. In blue (red) the energy levels for *N* electron on the bridge and one electron on the donor (acceptor). Only states within dashed lines are in the proper energy window to be involved in the ET. (c,d) Example of simulated electron transfer dynamics. (c) Charge on D, A (*n*_*i*_ = *n*_*i↑*_ + *n*_*i↓*_, *i* = *D*, *A*) and *χ n*_χ_ = *∑*_*i*∈χ_(*n*_*i↑*_ + *n*_*i↓*_). On χ we have subtracted the initial value, 4. (d) Corresponding spin polarization *p*_*i*_ = (*n*_*i↑*_ – *n*_*i↓*_)/*n*_*i*_ on A (black), D (red), and χ (blue). Inset: spin expectation values along the bridge axis 2⟨*S*_*z*,*i*_⟩ = *n*_*i↑*_ – *n*_*i↓*_ for bridge (blue), acceptor (black), and sum of the two (magenta). Detailed oscillations of *n*_*i*_ and *p*_*i*_ (*i* = 1, ..., 4) are shown in Figure S3. Simulation parameters: *t*/*U* = 0.0125, λ/*U* = 6.25 × 10^–4^, Γ/*U* = 2.5 × 10^–4^.

To investigate the role of correlations, we consider a prototypical many-body model and describe the chiral bridge χ by a half-filled chain of *N* orbitals and *N* electrons with a singlet ground state, characterized by
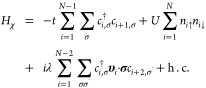
1Here *c*_*iσ*_^†^ (*c*_*iσ*_) creates (annihilates) an electron with spin σ on site *i* and *n*_*iσ*_=*c*_*iσ*_^†^*c*_*iσ*_. The first term is the hopping between neighboring sites, the second is the on-site Coulomb repulsion,^36,37^ and the last one is the spin–orbit coupling in form of a spin-dependent next-to-nearest neighbor hopping▲ . We follow the minimal model by Fransson,^21^ which does not target a specific system and hence yields general qualitative conclusions○. The vector **υ**_*i*_ is defined referring to a helix shape of the molecule with a single turn, radius *a*, pitch *c* and positions of the sites **r**_*i*_ = [*a* cos{(*i* – 1)2π/(*N* – 1)}, *a* sin{(*i* – 1)2π/(*N* – 1)}, (*i* – 1)*c*/(*N* – 1)], **υ**_*i*_ = **d**_*i*+1_ × **d**_*i*+2_ and **d**_*i*+*s*_ = (**r**_*i*_ – **r**_*i*+*s*_)/|**r**_*i*_ – **r**_*i*+*s*_|. Here, we assume *a* = *c* as in ref (20). Changing the enantiomer corresponds to the transformation (υ_*xi*_,υ_*yi*_,υ_*zi*_) → (-υ_*xi*_,υ_*yi*_,-υ_*zi*_). Donor (D) and Acceptor (A) are modeled by two additional sites at the two ends of the bridge [see Figure 1-(a) for *N* = 4], incoherently coupled to it (see below). A fast incoherent multistep ET was directly observed in many systems, such as the PXX-NMI_2_–NDI molecule displaying CISS.^34^ In eq 1 we assume degenerate orbitals, but this condition can be relaxed (see below). In addition, we do not explicitly consider the electrostatic energy contribution due to the charge separation from D to A^40^●.

The initial state after photoexcitation has two electrons in the singlet state on the two relevant donor orbitals, one each [blue oval in Figure 1-(a)]. In the absence of a coherent coupling between D and χ and before relaxation, we can trace out the state of the electron in the donor ground orbital and restrict to the moving electron, as demonstrated in Figure S1. Hence, we consider *many-body* eigenstates |ψ_μ_⟩ of the full supra-molecular Hamiltonian *H* = *H*_χ_ + *H*_*D*_ + *H*_*A*_ (*H*_*i*_ = *∑*_σ_*E*_*i*_*n*_*i*,σ_, *i* = *D*, *A*) for *N* + 1 = 5 electrons. These can be gathered into factorized |ψ_*k*_^*N*^σ_*D*/*A*_⟩ states with *N* electrons on the bridge and one either on the excited D orbital or on A, and |ψ_*j*_^*N*+1^⟩ states with *N* + 1 electrons delocalized on the bridge [Figure 1-(b), bottom]. The energies of these states are sketched in Figure 1-(b), with some of the |ψ_*j*_^*N*+1^⟩ lying in the energy window between the lowest-energy |ψ_*k*_^*N*^σ_*D*_⟩ and all the |ψ_*k*_^*N*^σ_*A*_⟩ states.

To drive ET, we consider jump operators *X*_*D*_=∑_σ_*c*_i=1,σ_^†^*c*_*Dσ*_+h.c. and *X*_*A*_=∑_σ_*c*_*Aσ*_^†^*c*_i=4,σ_+h.c., inducing spin-independent hopping from the donor excited orbital onto the bridge or from the bridge to the acceptor and we derive (see SI) the Redfield equation^41^ for the system density matrix ρ:
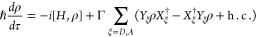
2

The first term of eq 2 describes the coherent evolution, while the second the incoherent transfer with *Y*_ξ_ = *∑*_μ,ν_|ψ_μ_⟩⟨ψ_ν_|⟨ψ_μ_|*X*_ξ_|ψ_ν_⟩*D*_μ,ν_. *D*_μ,ν_ are proportional to the bath spectral function  and to the Bose-Einstein factor  (for *E*_ν_ < *E*_μ_) or *n*(*x*) + 1 (for *E*_ν_ > *E*_μ_) evaluated at *x* = |*E*_ν_ – *E*_μ_|, i.e. the energy gap between eigenstates |ψ_μ_⟩ and |ψ_ν_⟩ of Hamiltonian *H*. Any dependence on specific properties of the bath is eliminated by considering the low-temperature limit and assuming energy independent  (wide-band approximation^42^), thus reducing *D*_μ,ν_ to Θ(*E*_ν_ – *E*_μ_), Θ being the Heaviside step-function. This implies that only the subset of |ψ_*j*_^*N*+1^⟩ states lying in the correct energy window [see Figure 1-(b)] is involved in the ET. We have checked that these assumptions do not significantly alter our results (see calculations with a typical Debye spectral density function^33,43^ in Figure S2). Other effects of temperature (e.g., a change of the initial state and of vibrations) are nontrivial and beyond the scope of this work.

Additional incoherent terms, modulating, e.g., hopping between different sites of the bridge, could be included in eq 2. However, they act on a much slower time scale compared to both the coherent dynamics (in particular in the (*N* + 1)-electron subspace) and the rates Γ (see Figure S4). Hence, we consider the associated relaxation dynamics on the bridge separately after the ET process (see below).

An example of the simulated charge separation dynamics and of the corresponding spin polarization is reported in Figure 1-(c,d). The system is initialized into ρ(0)=|ψ_0_^*N*^⟩⟨ψ_0_^*N*^|⊗(|↓_*D*_⟩⟨↓_*D*_|+|↑_*D*_⟩⟨↑_*D*_|)/2, i.e. the singlet ground state with *N* electrons on χ and a mixture of |*↑*⟩ and |*↓*⟩ in the donor excited state, as obtained from an excited singlet on D, after tracing out the electron in the ground orbital. Figure 1-(c) depicts that the charge *n*_*i*_ = *n*_*i↑*_ + *n*_*i↓*_ is completely transferred from D to A, after slightly populating (*N* + 1)-electron states on χ. In parallel, spin polarization *p*_*A*_ = 2*S*_*zA*_/*n*_*A*_ = (*n*_*A↑*_ – *n*_*A↓*_)/*n*_*A*_ quickly raises on A and then stabilizes at about 20% [black line in Figure 1-(d)], accompanied by a negative spin polarization distributed on the four sites of χ [*S*_*z*,*i*_ is the spin component along χ on site *i*].

Since we focus on the effect of correlations, we report the parameters in units of the Coulomb interaction *U*. In Figure 1-(c,d) *t* = 0.0125*U*, λ = 6.25 × 10^–4^*U* and Γ = 2.5 × 10^–4^*U*. By setting *U* = 4 eV, we get *t* = 0.05 eV, λ = 2.5 meV and Γ = 1 meV. These numbers for *t*(21,23,44,45) and λ^14,20,46^ are reasonable for many systems and in particular the small *t* is typical of DNA.^13,14,19,40,47^ The resulting time scale of ET is on the order of 10 ps.

## Insight into the Spin Polarization Mechanism

**Figure 2:**
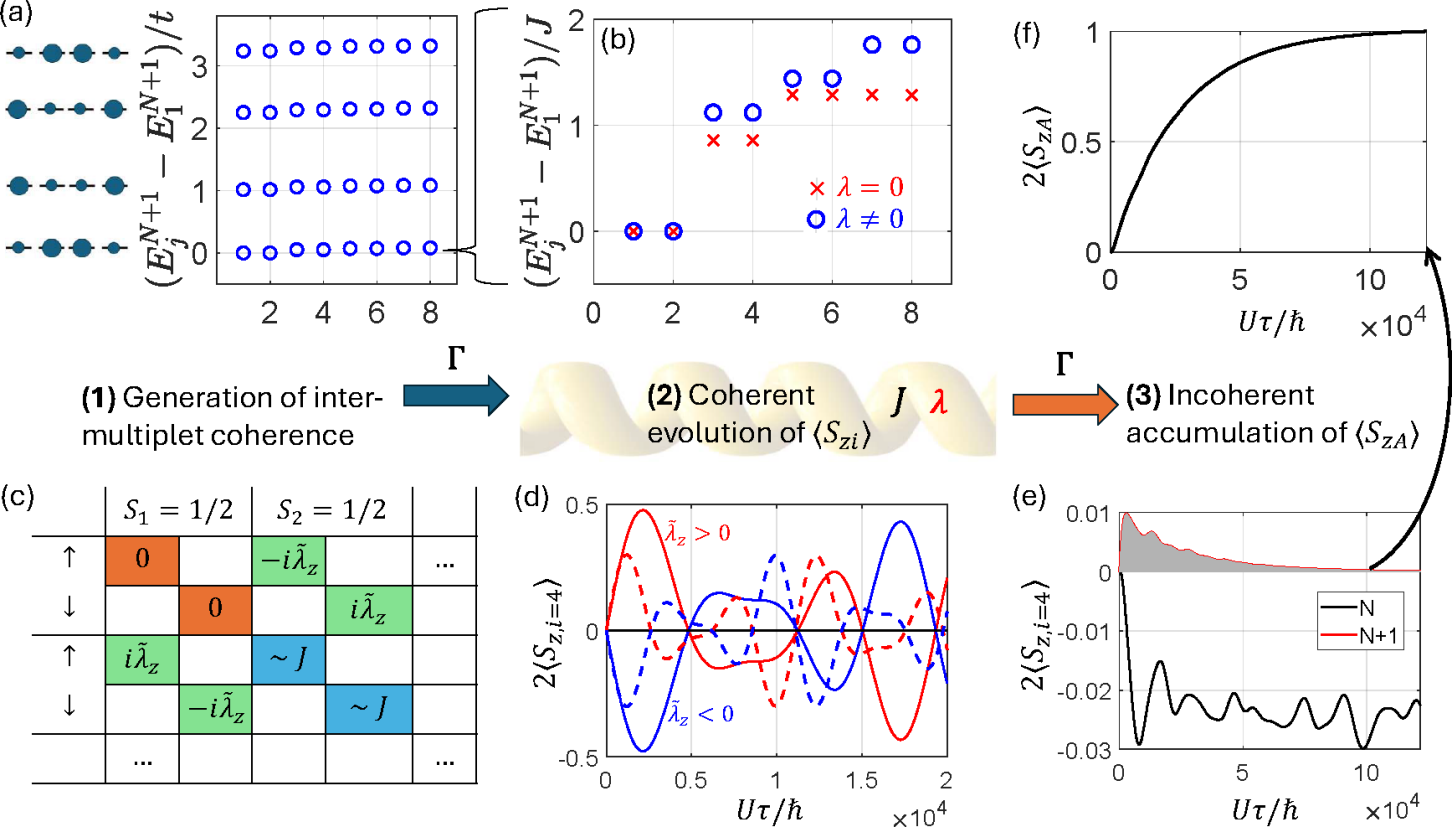
**Spin polarization mechanism.** (a) Lowest-energy many-body states |ψ_*j*_^*N*+1^⟩ (*j* = 1, ..., 32) within the (*N* + 1)-electron subspace. On the left, a sketch of the charge distribution of the (*N* + 1) = 5 delocalized electrons on χ, calculated as the expectation value of *n*_*i*_. (b) Zoom on the lowest energy block of the (*N* + 1)-electron subspace. (c) Scheme of nonzero populations (red and blue) and coherences (green) in the density matrix generated by the jump operator *X*_*D*_ from D to χ and corresponding matrix elements of the Hamiltonian (symbols), explicitly shown for the two lowest energy doublets of panel (b) with . In this panel, to highlight the effect of SOC, we use the eigenbasis of *H* without SOC (i.e., λ = 0). (d) Time evolution of 2⟨*S*_*z*,*i*=4_⟩ for an initial state prepared into *X*_*D*_ρ(0)*X*_*D*_^†^ (with population only in the lowest energy block of Figure 2-(a), i.e. |ψ_*j*_^*N*+1^⟩, *j* = 1, ..., 8) and using *t*/*U* = 0.0125, λ_*z*_/*U* = 0.0005. With the basis used in panel (c), the amplitude of the oscillations is proportional the real part of intermultiplet coherences. Results for the two enantiomers (corresponding to opposite ) are represented by different colors, while dashed lines are obtained by halving correlations (i.e., using *t*/*U* = 0.025, λ_*z*_/*U* = 0.001). (e) Full time evolution of 2⟨*S*_*z*,*i*=4_⟩ including both coherent and incoherent dynamics in eq 2 and separated into contributions of states with either *N* or *N* + 1 electrons on χ. The latter is proportional to the derivative of *S*_*zA*_ accumulated on the acceptor (f), which can therefore be obtained from the shaded area in (e).

To understand the mechanism building up a spin polarization on A we need to focus on the (*N* + 1) – electron many-body states |ψ_*j*_^*N*+1^⟩ reported in Figure 2-(a,b). The lowest energy |ψ_*j*_^*N*+1^⟩ (*j* = 1, ..., 32) are organized in four blocks with different charge distribution (sketched on the left), split by ∼ *t* [Figure 2-(a)]. Each block displays a similar spin structure, consisting of two total-spin doublets *S* = 1/2 and a higher-energy quartet *S* = 3/2, split by the exchange *J* = 4*t*^2^/*U* [Figure 2-(b)]. SOC further splits the quartet into two Kramer’s pairs (crosses vs circles). Note that, due to the energy dependent term *D*_μ,ν_ in eq 2, only some |ψ_*j*_^*N*+1^⟩ states participate in the ET [we consider the lowest energy block of Figure 2-(a)].

The ingredients contributing to the rise of *p*_*A*_ are depicted in Figure 2-(c-e). First **(1)**, the incoherent jump operator *X*_*D*_ generates both populations and *coherences*□ [colors in panel (c)] between different multiplets of Figure 2-(b). Then, the combined effect of *J* (which splits different multiplets) and λ (which mixes them) as sketched in Figure 2-(c)■, yields coherent oscillations **(2)** of the local spin polarization on different sites, and in particular of *S*_*z*,*i*=4_ [Figure 2-(d)]. Different enantiomers yield opposite *S*_*zi*_ (red/blue curves). Spin polarization originates from the small SOC, whose effect on the coherent evolution *can become significant in the presence of a similar energy scale*, provided by *J*. The amplitude of these oscillations is proportional to the intermultiplet coherence, which increases for small *J* ∝ *t*^2^/*U*, i.e. strong correlations.

The coherent oscillations of ⟨*S*_*zi*_⟩ are necessary but not sufficient to explain the spin polarization *accumulated* on A. The last ingredient **(3)** is represented by the incoherent terms in the Redfield eq 2. Hence, we derive (see SI) the variation of charge on A for an elementary time step according to eq 2. We find
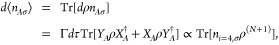
3i.e. the variation of ⟨*n*_*Aσ*_⟩ is proportional to *n*_*i*=4,σ_ within the (*N* + 1)-electron subspace. Hence, ⟨*S*_*zA*_⟩ is proportional to the time integral of ⟨*S*_*z*,*i*=4_⟩ within the (*N* + 1)-electron subspace. Note that ⟨*S*_*z*,*i*=4_⟩ oscillates, but it is *always positive* within the (*N* + 1)-electron subspace [Figure 2-(e)]. Hence, its integral (∝⟨ *S*_*zA*_⟩) is a monotonic increasing function [Figure 2-(f)], resulting from the interplay between coherent and incoherent dynamics on similar time scales◇. The sign of the spin polarization does not depend on the specific choice of the parameters *t*, *U* and Γ, but is reversed by changing the molecular chirality. This effect on spin polarization practically vanishes if correlations (responsible of the multiplet structure of the |ψ_*j*_^*N*+1^⟩ states) are neglected.

## Numerical Simulations

**Figure 3:**
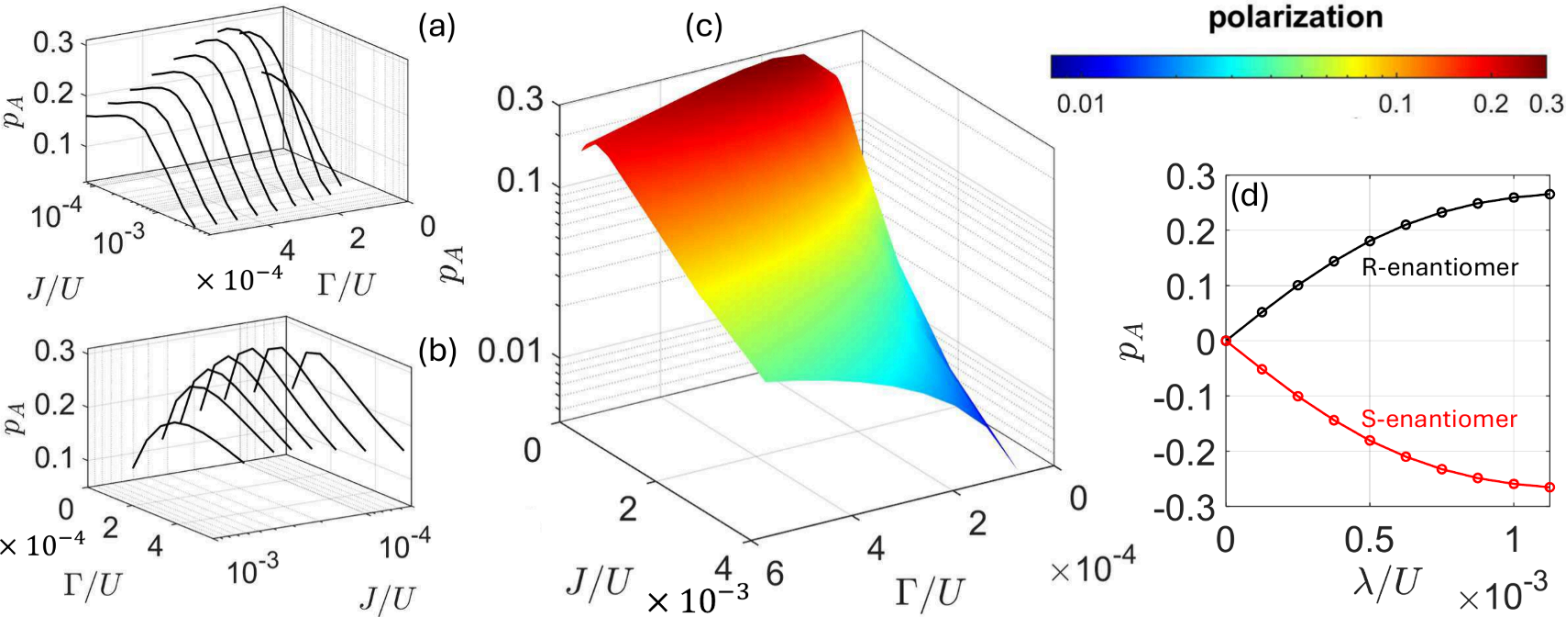
**Dependence of spin polarization on model parameters**. (a-c) Acceptor spin polarization *p*_*A*_ = (*n*_*A↑*_ – *n*_*A↓*_)/(*n*_*A↑*_ + *n*_*A↓*_) as a function of *J* = 4*t*^2^/*U*, and of Γ with cuts along *J* (a) and Γ (b) for fixed λ = 6.25 × 10^–4^*U* and the whole colormap (c). (d) Dependence of *p*_*A*_ on λ, for the two opposite enantiomers [related by the transformation (υ_*xi*_,υ_*yi*_,υ_*zi*_) → (-υ_*xi*_,υ_*yi*_,-υ_*zi*_) in eq 1], using *t*/*U* = 0.0125 and Γ/*U* = 3.125 × 10^–4^.

Having clarified the spin polarization mechanism, we investigate the dependence of *p*_*A*_ on the model parameters. Results are summarized in Figure 3. Panels (a-c) show the behavior of *p*_*A*_ as a function of the incoherent transfer rate Γ and of the exchange *J*, for fixed λ = 6.25 × 10^–4^*U*. We note a maximum, both as a function of *J* = 4*t*^2^/*U* and of Γ, more evident in the cuts reported in panels (a) and (b). This nontrivial dependence highlights the complex interplay between coherent and incoherent dynamics. Remarkably, *p*_*A*_ vanishes for vanishing correlations (i.e., large *t*/*U*) and reaches its maximum for small *J*. The highest spin polarization ∼30% is achieved for *J*/*U* = 1.56 × 10^–4^ (corresponding to *t*/*U* ≈ 6.25 × 10^–3^) and intermediate Γ ≈ 2 × 10^–4^*U*. This condition *J* ∼ Γ for the optimal spin polarization is consistent with findings of refs (33, 48). in a simplified model which does not include the degrees of freedom of the bridge. Note, however, that the meaning of *J* is very different in refs (33, 48) because here it represents the exchange interaction within the bridge yielding entangled many-body states, while there it accounts for the coupling between the transferred electron and the one remaining on D.

Note that *p*_*A*_ also depends on the number of |ψ_*j*_^*N*+1^⟩ states participating in the ET and it strongly decreases by including higher energy blocks in Figure 2-(a). Figure 3-(d) reports the increase of |*p*_*A*_| with λ, with opposite sign for different enantiomers.

**Figure 4:**
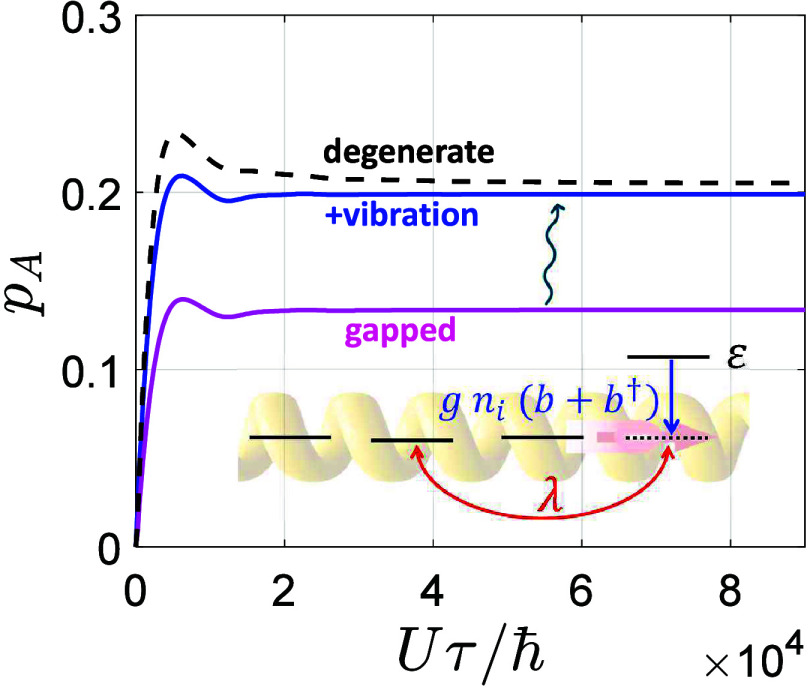
**Nonequivalent orbitals and polarons**. Effect on the acceptor spin polarization *p*_*A*_ of introducing an energy gap ε in *H*_χ_ on the fourth site (magenta line) and of adding a local vibration (blue), thus approaching the degenerate situation (black dashed curve). Inset: sketch of the minimal model we consider (with the Fermion-boson coupling in blue and details in the SI), with *t*/*U* = 0.0125, λ/*U* = 6.25 × 10^–4^, Γ/*U* = 2.5 × 10^–4^, ε/*U* = 0.0375, *ℏω*_0_/*U* = 0.0125, *g*/*U* = 0.01625, up to 9 bosons included.

Energy gaps between orbitals^40^ would possibly yield more localized eigenstates, affecting ET. We investigate this and find a significant spin polarization even in the presence of a sizable gap ε ≫ λ between orbitals (Figure 4, magenta line). Moreover, strong electron coupling with local vibrations which can occur in these systems^49^ leads to a renormalization of the orbital energies which can effectively reduce ε, and hence result in spin polarization dynamics close to Figure 1. Although an extensive study on polarons is beyond the scope of this work, we show this by the simulations of Figure 4, where an energy gap ε on one site with respect to the others and the strong coupling with a local vibration are explicitly included (see SI).

## Discussion

The final state in the simulations above is a superposition/mixture of various many-body eigenstates |ψ_*k*_^*N*^σ_*A*_⟩ and is expected to undergo thermal relaxation. This will occur on a time scale longer than ET, but slower than EPR experiments,^34^ > 10–50 ns. Therefore, EPR typically probes the state of D−χ – A with two unpaired electrons (one on D and one on A) *after relaxation* of χ into its singlet ground state. Hence, we perform simulations starting with a singlet electron pair on D and including thermal relaxation, modeled via a rate-equation in the system eigenbasis (SI).◆ We obtain complete relaxation of the bridge into its singlet ground state (see Figure S5), with its transient spin polarization redistributed among D and A. The resulting state of the DA radical pair has opposite spin polarization on D and A. This state is different from the singlet typical for spin-correlated radical pairs linked through nonchiral bridges, and hence yields a different EPR spectrum,^7,30^ as observed.^34^ Since our conclusions hold for a wide range of parameters (see Figure 3) we expect to detect CISS in a similarly broad class of systems, besides ref (34).

Another point concerns the dependence on the length of χ. Two different situations could occur: (i) ET takes place in two incoherent steps (as above) from D to χ and from χ to A, interleaved by the coherent evolution of the bridge many-body states. Then, a longer bridge with similar parameters *J* ∼ λ will exhibit a qualitatively similar evolution. We checked this by computing the dynamics for *N* = 6, finding a spin polarization comparable to that obtained with *N* = 4 (Figure S6). (ii) ET occurs as a sequential multistep incoherent process through different parts of a longer bridge. In that hypothesis, the process can be approximated as a concatenation of steps in which the final spin state becomes the initial state of the next one. Hence, spin polarization can accumulate in each step. For instance, restarting from *p*_*A*_ = 0.20 we get 0.27 and 0.29 in the following two steps [Figure S6]. These numbers depend on the parameters, but indicate that in a sequential multistep ET spin polarization can increase with the length of the bridge, as observed in conductance measurement on thick films of DNA and oligopeptides.^1^

Summarizing, we report the first microscopic model for CISS in electron transfer through chiral molecules, with the explicit inclusion of the bridge degrees of freedom that play an active role in the electron spin polarization. Based on experimental evidence of spin polarization even on short chiral chains, we build a minimal model system on a few sites and we pinpoint the essential ingredients for achieving a sizable spin polarization even in the presence of a small spin–orbit coupling. These are (i) strong electron–electron correlations giving rise to many-body states split by the exchange interaction; (ii) an interplay of coherent (exchange + spin–orbit) dynamics on the chiral bridge and incoherent hopping from the donor or onto the acceptor; (iii) relaxation of the chiral bridge to establish the long-term spin polarization observed experimentally.

## Supporting Information Available

Definition of the spin–orbit coupling, simulations starting from a singlet spin pair on the donor, details on the Redfield equation at finite temperature, simulations including bath spectral density, electron transfer dynamics on different sites, derivation of spin polarization accumulated on A, coupling with vibrations, relaxation, length dependence (PDF)

## Footnotes

▲ is a short-hand notation (see, e.g., ref.^14^) for υ_*ix*_(*c*_*i*,↑_^†^*c*_*i*+2,↓_+*c*_*i*,↓_^†^*c*_*i*+2,↑_)-*iυ*_*iy*_(*c*_*i*,↑_^†^*c*_*i*+2,↓_-*c*_*i*,↓_^†^*c*_*i*+2,↑_)+υ_*iz*_(*c*_*i*,↑_^†^*c*_*i*+2,↑_-*c*_*i*,↓_^†^*c*_*i*+2,↓_).

○ Hamiltonian eq 1 ensures the presence of two channels for electron transfer and hence opens the possibility of a spin polarization. Indeed, no spin polarization arises in the presence of only nearest-neighbor interactions both in the hopping and SOC terms of eq 1, consistent with reports for transport in a two-terminal setup.^13,38,39^ An even number of orbitals on the bridge ensures that all the sites take part in both channels.

● This effect is implicitly included in the hierarchy of the many-body states.

□ Off-diagonal elements of ρ.

■ For illustrative purposes we consider here only λ_*z*_ = *λυ*_*iz*_, but all other simulations are performed with the hole **υ**_*i*_.

◇ Note that the three steps **(1–3)** are all interconnected and occur simultaneously in the dynamics.

◆ For simplicity, we only include the υ_*z*_ contribution to the SOC. We consider modulation of leading one-body terms in the Hamiltonian. Moreover, to allow for relaxation from states with *∑*_*i*∈χ_*S*_*z*,*i*_ ≠ 0 into the singlet ground state of χ, we include a modulation of the coupling of the bridge end spins with the two spins on D and A. Indeed, modulation of nearest-neighbor hopping and orbital energies involve rank-0 operators on the bridge, which preserve both its total spin and its *z* component.
