# Supplementary material for: Many-Body Models for Chirality-Induced Spin Selectivity in Electron Transfer
Source: Nano Lett. 2024 Sep 22;24(39):12133–9. doi: 10.1021/acs.nanolett.4c02912 (PMC11450995; doi:10.1021/acs.nanolett.4c02912)
Supplement: Supplementary file 1 — nl4c02912_si_001.pdf [file nl4c02912_si_001.pdf]

Supporting Information to Many-Body Models for Chirality-Induced Spin Selectivity in Electron Transfer

Alessandro Chiesa,[1,2,3] Elena Garlatti,[1,2,3] Matteo Mezzadri,[1,2] Leonardo Celada,[1,2] Roberta Sessoli,[4,3] Michael R. Wasielewski,[5] Robert Bittl,[6] Paolo Santini,[1,2,3] and Stefano Carretta[1,2,3,*]

[1]*Università di Parma, Dipartimento di Scienze Matematiche, Fisiche e Informatiche, I-43124 Parma, Italy*

[2]*INFN–Sezione di Milano-Bicocca, gruppo collegato di Parma, 43124 Parma, Italy*

[3]*Consorzio Interuniversitario Nazionale per la Scienza e Tecnologia dei Materiali (INSTM), I-50121 Firenze, Italy*

[4]*Dipartimento di Chimica "U. Schiff" (DICUS), Università degli Studi di Firenze, I-50019 Sesto Fiorentino (FI), Italy*

[5]*Department of Chemistry, Center for Molecular Quantum Transduction, and Institute for Sustainability and Energy at Northwestern, Northwestern University, Evanston, Illinois 60208-3113, United States*

[6]*Freie Universität Berlin, Fachbereich Physik, Berlin Joint EPR Lab, D-14195 Berlin, Germany*

I. DEFINITION OF THE SPIN-ORBIT COUPLING

The form of the spin-orbit coupling (SOC) used in Eq. (1) of the main text

$$H_{SOC} = i\lambda \sum_{i=1}^{N-2} \sum_{\sigma\sigma'} c^{\dagger}_{i,\sigma} \boldsymbol{\upsilon}_i \cdot \boldsymbol{\sigma}\, c_{i+2,\sigma'} + \text{h.c.} \tag{S1}$$

is a short-hand notation (see, e.g., Ref. [S1]) for

$$\begin{aligned} H_{SOC} = i\lambda\Big[&\upsilon_{ix}\left(c^{\dagger}_{i,\uparrow}c_{i+2,\downarrow} + c^{\dagger}_{i,\downarrow}c_{i+2,\uparrow}\right) \\ &-i\upsilon_{iy}\left(c^{\dagger}_{i,\uparrow}c_{i+2,\downarrow} - c^{\dagger}_{i,\downarrow}c_{i+2,\uparrow}\right) \\ &+\upsilon_{iz}\left(c^{\dagger}_{i,\uparrow}c_{i+2,\uparrow} - c^{\dagger}_{i,\downarrow}c_{i+2,\downarrow}\right)\Big] + \text{h.c.} \end{aligned} \tag{S2}$$

$\boldsymbol{\upsilon}_i$ is defined referring to a helix shape of the molecule with a single turn, with radius a, pitch c and positions of the sites

$$\mathbf{r}_i = [a\cos\{(i-1)2\pi/(N-1)\}, a\sin\{(i-1)2\pi/(N-1)\}, (i-1)c/(N-1)]. \tag{S3}$$

Then, $\boldsymbol{\upsilon}_i = \mathbf{d}_{i+1} \times \mathbf{d}_{i+2}$ and $\mathbf{d}_{i+s} = (\mathbf{r}_i - \mathbf{r}_{i+s})/|\mathbf{r}_i - \mathbf{r}_{i+s}|$ as in [S2]. With these definitions, changing the enantiomer corresponds to the transformation $(\upsilon_{xi}, \upsilon_{yi}, \upsilon_{zi}) \rightarrow (-\upsilon_{xi}, \upsilon_{yi}, -\upsilon_{zi})$.

The next-to-nearest neighbor SOC [S2–S4] is a minimal choice which ensures in Hamiltonian (1) of the main text the presence of two channels for electron transfer and hence opens the possibility of a spin polarization. Indeed, we have checked that no polarization arises in presence of only nearest-neighbor interactions both in the hopping and SOC terms of Eq. (1), consistent with reports for transport in a two-terminal setup [S5–S7].

We stress that the mechanism we present for spin polarization does not depend on this choice. For instance, analogous results are obtained by using a nearest-neighbor spin-orbit coupling in presence of nearest and next-to-nearest neighbor spin-independent hopping. As an example, a spin polarization larger than 0.2 is obtained by setting a next-to-nearest neighbor hopping and a nearest-neighbor spin-orbit coupling of $6.25 \times 10^{-4}\, U$ (z component only, for simplicity), with $\Gamma = 6.25 \times 10^{-5}\, U$ and $t = 0.0125\, U$.

* stefano.carretta@unipr.it

II. SIMULATIONS STARTING FROM A SINGLET PAIR ON THE DONOR

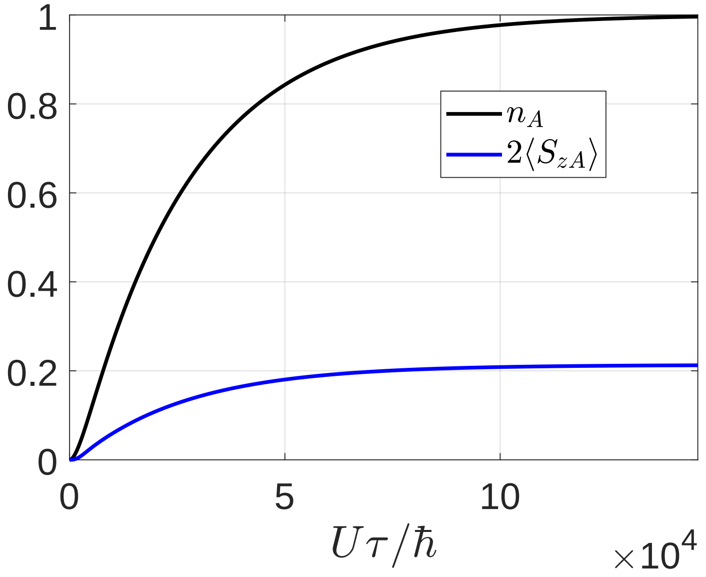

Figure S1: **Electron transfer dynamics starting from a singlet on D**. Time evolution of charge (black) and of $2\langle S_z\rangle$ (blue) for an initial pure state consisting of a photo-excited spin singlet on the donor and a singlet on χ. Results perfectly match those reported in Fig. 1-(c,d) of the main text, after tracing out the electron sitting in the donor ground state. Parameters of the simulations: $t/U = 0.0125$, $\lambda/U = 6.25 \times 10^{-4}$, $\Gamma/U = 2.5 \times 10^{-4}$.

III. REDFIELD EQUATION FOR ELECTRON TRANSFER

To describe the electron-transfer (ET) dynamics, we consider an interaction between the system and the bath of the form

$$H_{SB} = \sum_r \sum_{\nu=D,A} \kappa_{r,\nu}(X_\nu + X_\nu^\dagger)\,(a_{r,\nu} + a_{r,\nu}^\dagger) \tag{S4}$$

with the operators $X_D = \sum_\sigma c_{1\sigma}^\dagger c_{D\sigma}$ and $X_A = \sum_\sigma c_{A\sigma}^\dagger c_{i=4,\sigma}$ inducing electron hopping from the donor excited orbital onto the bridge or from the bridge to the acceptor, respectively. Here $a_{r,\nu}$ is the bosonic annihilation operator for the r-th mode of the bath coupled with a strength $\kappa_{r,\nu}$ to either X_D or X_A. X_ν are rank-0 fermionic operators which do not affect the spin of the transferred electron. For simplicity, we do not include further coupling terms between the system and the bath.
We consider temperatures much smaller than the energy gaps driving ET and we describe the time evolution of the system density matrix ρ by the Redfield Eq. (2) of the main text [S8]. This correctly accounts for both population and coherences to order Γ [S8], and we have checked that that positivity of ρ is granted in our simulations within $\sim 10^{-5}$.

A. Redfield equation at finite temperature

We recall the Redfield equation used in the main text:

$$\hbar\frac{d\rho}{d\tau} = -i[H,\rho] + \Gamma \sum_{\xi=D,A} \left(Y_\xi \rho X_\xi^\dagger - X_\xi^\dagger Y_\xi \rho + \text{h.c.} \right). \tag{S5}$$

Note that we have not to applied the secular approximation [S8], given the comparable magnitude of the incoherent rates and of the smallest energy gaps in the molecular spectrum. In the main text we have considered the low-temperature and wide-band limits to Eq. (S5). Here we take the more general expression for $D_{\mu\nu}$ [S8], namely

$$D_{\mu\nu} \propto \begin{cases} n(E_\mu - E_\nu)\,\mathfrak{I}(E_\mu - E_\nu) & \text{for } E_\nu < E_\mu \\ [n(E_\nu - E_\mu) + 1]\,\mathfrak{I}(E_\nu - E_\mu) & \text{for } E_\nu > E_\mu \end{cases} \tag{S6}$$

accounting for both absorption and emission processes in the transition $|\psi_\nu\rangle \rightarrow |\psi_\mu\rangle$. Here $n(\omega) = 1/\left(e^{\omega/k_B T} - 1\right)$ is the Bose-Einstein factor and $\mathfrak{I}(\omega)$ is the bath spectral density.
In Fig. S2 we report simulations of the electron transfer process performed using a typical

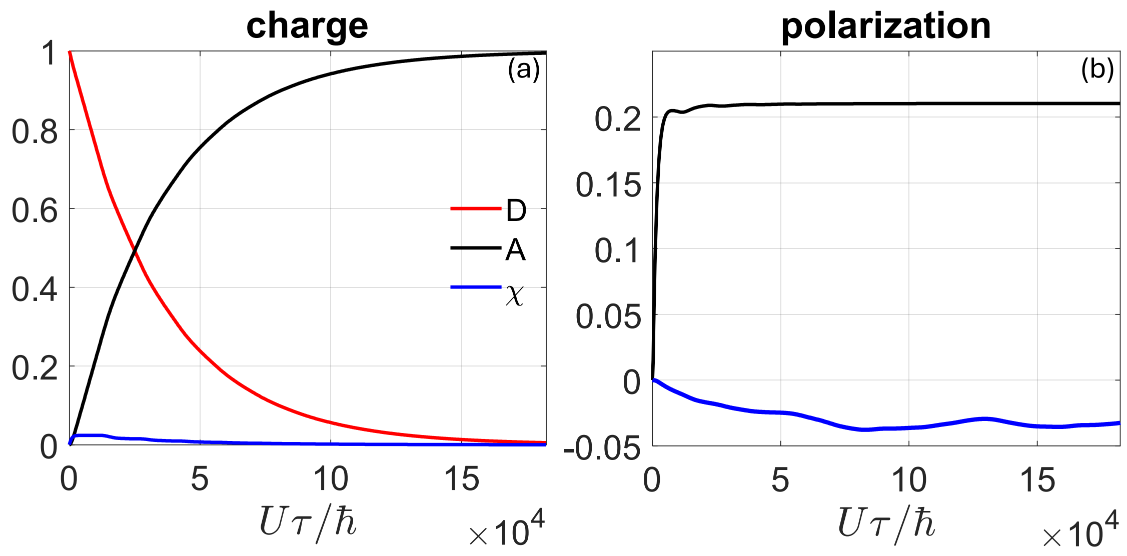

Figure S2: (a) Charge on D, A and χ (having subtracted its initial value, 4) and (b) corresponding spin polarization on the donor (red), acceptor (black) and on the bridge (blue) obtained by numerically integrating the Redfield equation at 85 K and with a Debye spectral density function of the bath, with $t/U = 0.0125$, $\lambda/U = 6.25 \times 10^{-4}$.

Debye spectral density function $\mathfrak{J}(\omega) \propto \omega\,\omega_c/(\omega^2 + \omega_c^2)$ [S9, S10] at 85 K, as in recent experimental observations [S11]. Results are equivalent to those reported in the main text using the low-temperature and wide-band approximations [i.e. $D_{\mu\nu} = \Theta(E_\nu - E_\mu)$], apart from a renormalization of the incoherent rates. This is reasonable, since different blocks of states involved in the electron transfer are rather close in energy compared to the cutoff energy $\omega_c \sim 0.1$ eV and gaps between different blocks (Fig. 2a of the main text) are large compared to $k_B T$.

IV. ELECTRON TRANSFER DYNAMICS ON DIFFERENT SITES

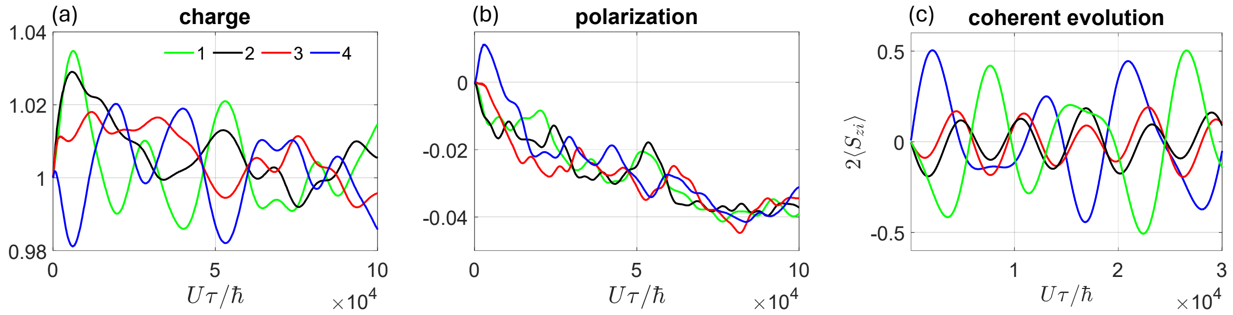

Figure S3: **Oscillation of local observables during the ET**. (a) Charge $n_i = n_{i\uparrow} + n_{i\downarrow}$ and (b) polarization $p_i = (n_{i\uparrow} - n_{i\downarrow})/(n_{i\uparrow} + n_{i\downarrow})$ obtained by numercally solving the Redfield Eq. (2) of the main text. (c) $2\langle S_{zi}\rangle = (n_{i\uparrow} - n_{i\downarrow})$ in presence of only the coherent Hamiltonian evolution on the $(N+1)-$electron subspace for an initial state prepared into $X_D \rho(0) X_D^\dagger$ (with population only in the lowest energy block of Fig. 1-(a), i.e. $|\psi_j^{N+1}\rangle$, $j = 1, ..., 8$), analogously to Fig. 2(d) of the main text. Parameters of the simulations: $t/U = 0.0125$, $\lambda/U = 6.25 \times 10^{-4}$, $\Gamma/U = 2.5 \times 10^{-4}$.

V. ELECTRON TRANSFER INCLUDING INCOHERENT DYNAMICS ON THE BRIDGE

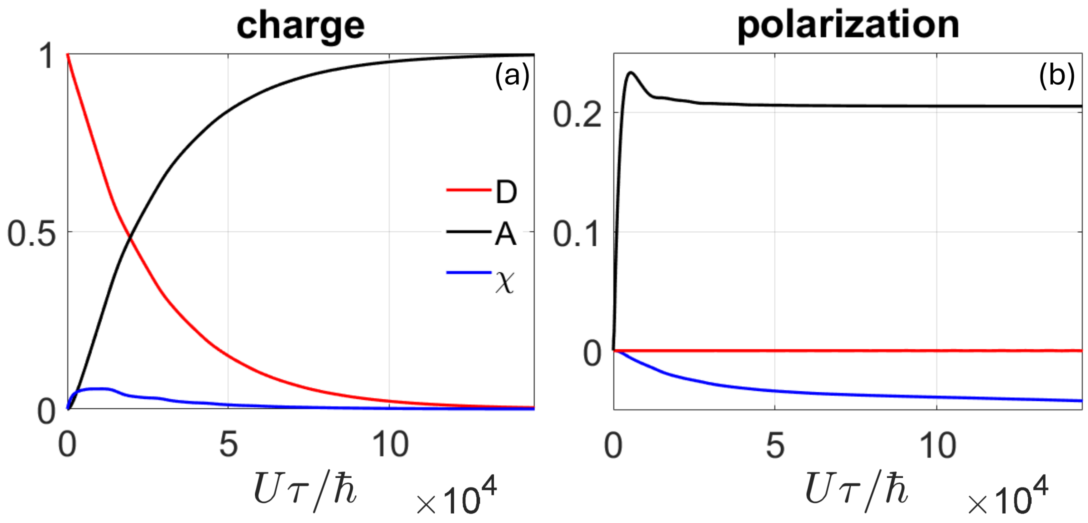

Figure S4: **ET including incoherent hopping within the bridge**. (a) Charge on D, A and χ (having subtracted its initial value, 4). (b) Corresponding spin polarization on the acceptor $p_A = (n_{A,\uparrow} - n_{A,\downarrow})/(n_{A,\uparrow} + n_{A,\downarrow})$ (black), the donor (red), and on the bridge (blue). Simulation parameters: $t/U = 0.0125$, $\lambda/U = 6.25 \times 10^{-4}$, $\Gamma/U = 2.5 \times 10^{-4}$ for all incoherent hopping rates (in-, outward and intra-bridge).

VI. DERIVATION OF THE SPIN POLARIZATION ACCUMULATED ON A

To derive the expression reported in Eq. (3) of the main text, we can expand X_A on the basis of the system eigenvectors as follows:

$$X_A = \sum_k^{(N)} \sum_j^{(N+1)} \sum_\sigma |\psi_k^N \sigma_A\rangle \langle \psi_k^N \sigma_A| \, c^\dagger_{A\sigma} c_{i=4,\sigma} \, |\psi_j^{N+1}\rangle \langle \psi_j^{N+1}| \,. \tag{S7}$$

where we have omitted $c^\dagger_{i=4,\sigma} c_{A\sigma}$ terms, assuming the energy is always decreasing when moving an electron from the bridge to the acceptor. Therefore, $X_A = Y_A$ in Eq. (2) of the main text. Then, we evaluate $d\langle n_{A\sigma}\rangle$ by separately considering the three terms of the Redfield equation $2X_A \rho X_A^\dagger$ (i), $X_A^\dagger X_A \rho$ (ii) and $\rho X_A^\dagger X_A$ (iii). The first one gives

$$\begin{aligned}
&\mathrm{Tr}\left[X_A \rho(\tau) X_A^\dagger n_{A\sigma}\right] = \qquad\qquad (S8)\\
&\sum_{k''\sigma''} \langle \psi_{k''}^N \sigma''_A| \left(\sum_{k,k'} \sum_{j,j'} \sum_{\sigma''',\sigma'} |\psi_k^N \sigma'''_A\rangle \langle \psi_k^N \sigma'''_A| \, c^\dagger_{A\sigma'''} c_{i=4,\sigma'''} \, |\psi_j^{N+1}\rangle \langle \psi_j^{N+1}| \, \rho \, |\psi_{j'}^{N+1}\rangle \right.\\
&\left. \langle \psi_{j'}^{N+1}| \, c^\dagger_{i=4,\sigma'} c_{A\sigma'} \, |\psi_{k'}^N \sigma'_A\rangle \langle \psi_{k'}^N \sigma'_A| \right) n_{A\sigma} \, |\psi_{k''}^N \sigma''_A\rangle \\
&= \sum_{k\sigma} \sum_{j,j'} \langle \psi_k^N \sigma_A| \, c^\dagger_{A\sigma} c_{i=4,\sigma} \, |\psi_j^{N+1}\rangle \langle \psi_j^{N+1}| \, \rho \, |\psi_{j'}^{N+1}\rangle \langle \psi_{j'}^{N+1}| \, c^\dagger_{i=4,\sigma} c_{A\sigma} \, |\psi_k^N \sigma_A\rangle \,.
\end{aligned}$$

We now note that $c_{A\sigma} |\psi_k^N \sigma_A\rangle = |\psi_k^N\rangle$ and hence we can reduce the above expression to operators acting only on the bridge:

$$\mathrm{Tr}\left[X_A \rho(\tau) X_A^\dagger\right] = \sum_k \sum_{j,j'} \langle \psi_k^N| \, c_{i=4,\sigma} \, |\psi_j^{N+1}\rangle \langle \psi_j^{N+1}| \, \rho \, |\psi_{j'}^{N+1}\rangle \langle \psi_{j'}^{N+1}| \, c^\dagger_{i=4,\sigma} \, |\psi_k^N\rangle \tag{S9}$$

Exploiting completeness relations in the $(N+1)-$electron subspace $\sum_j |\psi_j^{N+1}\rangle \langle \psi_j^{N+1}| = I$ and defining $\rho^{N+1} \equiv \sum_{jj'} |\psi_j^{N+1}\rangle \langle \psi_j^{N+1}| \, \rho \, |\psi_{j'}^{N+1}\rangle \langle \psi_{j'}^{N+1}|$, we get

$$\begin{aligned}
\mathrm{Tr}\left[X_A \rho(\tau) X_A^\dagger\right] &= \sum_k \langle \psi_k^N| \, c_{i=4,\sigma} \; \rho^{N+1} \; c^\dagger_{i=4,\sigma} \, |\psi_k^N\rangle \\
&= \mathrm{Tr}\left[c_{i=4,\sigma} \; \rho^{N+1} \; c^\dagger_{i=4,\sigma}\right] \\
&\equiv \mathrm{Tr}\left[\rho^{N+1} \; c^\dagger_{i=4,\sigma} c_{i=4,\sigma}\right] = \langle n_{i=4,\sigma}\rangle_{N+1}.
\end{aligned} \tag{S10}$$

The other two terms (ii,iii) of the Redfield equation do not contribute to $d\langle n_{A\sigma}\rangle$. Indeed,

$$X_A^\dagger X_A = \sum_{j,j'} \sum_k \sum_\sigma |\psi_j^{N+1}\rangle \langle \psi_j^{N+1}| \, c^\dagger_{i=4,\sigma} \, |\psi_k^N\rangle \langle \psi_k^N| \, c_{i=4,\sigma} \, |\psi_{j'}^{N+1}\rangle \langle \psi_{j'}^{N+1}| \tag{S11}$$

only operates within the $(N+1)-$electron subspace. Conversely, $n_{A\sigma}$ is non-zero only on states belonging to the $N-$electron subspace. Hence

$$\mathrm{Tr}\left[X_A^\dagger X_A \rho \, n_{A\sigma}\right] = \sum_k \langle\psi_k^N|\, X_A^\dagger X_A \rho \,|\psi_k^N\rangle = 0 \tag{S12}$$

for orthogonality of the two groups of states $|\psi_j^{N+1}\rangle$ and $|\psi_k^N\rangle$. An analogous reasoning holds for the last term $\rho X_A^\dagger X_A$. Therefore, the only contribution to $d\langle n_{A\sigma}\rangle$ is from Eq. (S10), and we obtain $d\langle n_{A\sigma}\rangle \propto \langle n_{i=4,\sigma}\rangle_{N+1}$.

VII. COUPLING WITH VIBRATIONS AND DERIVATION OF THE EFFECTIVE HAMILTONIAN

We consider the toy model sketched in Fig. 4 of the main text, which extends the bridge Hamiltonian H_χ [Eq. (1)] as follows:

$$H_{\chi,v} = H_\chi + \varepsilon \sum_\sigma c_{i\sigma}^\dagger c_{i\sigma} + \ \hbar\omega_0 \left(b^\dagger b + \frac{1}{2}\right) + H_1, \tag{S13}$$

with $n_i = \sum_\sigma c_{i\sigma}^\dagger c_{i\sigma}$, and $b^\dagger(b)$ bosonic creation (annihilation) operators of a mode of energy $\hbar\omega_0$, locally coupled to site i. We recall that

$$H_\chi = -t\sum_{i=1}^{N-1}\sum_\sigma c_{i,\sigma}^\dagger c_{i+1,\sigma} + U\sum_i n_{i\uparrow}n_{i\downarrow} + \ i\lambda\sum_{i=1}^{N-2}\sum_{\sigma\sigma'} c_{i,\sigma}^\dagger \, \boldsymbol{\upsilon}_i\cdot\boldsymbol{\sigma}\, c_{i+2,\sigma'} + \text{h.c.}, \tag{S14}$$

and the *local* coupling term is given by

$$H_1 = g\left(b + b^\dagger\right) n_i. \tag{S15}$$

To derive an effective Hamiltonian in which H_1 is removed, we apply the Schrieffer-Wolff transformation with the following ansatz [S12, S13]:

$$S = \gamma\, g \sum_{\sigma=\uparrow,\downarrow} c_{i,\sigma}^\dagger c_{i,\sigma}(b^\dagger - b), \tag{S16}$$

where γ is a real coefficient to be determined. We immediately observe that $S^\dagger = -S$ and hence $e^S = \left(e^{-S}\right)^\dagger$.

We then consider the transformed operators $\tilde{b} = e^S b e^{-S}$ and $\tilde{c}_{i,\sigma'} = e^S c_{i,\sigma'} e^{-S}$. After some

algebra [S12, S13], we get

$$\begin{aligned}\tilde{b} &= e^{S}\, b\, e^{-S} \\ &= b + [S,b] + \frac{1}{2!}[S,[S,b]] + ... \\ &= b + \gamma g \sum_{\sigma=\uparrow\downarrow} c^{\dagger}_{i,\sigma} c_{i,\sigma}[b^{\dagger} - b, b] + \frac{1}{2!}[S,[S,b]] + ... \\ &= b - \gamma g \sum_{\sigma=\uparrow\downarrow} c^{\dagger}_{i,\sigma} c_{i,\sigma} + \frac{1}{2!}[S, -\gamma g \sum_{\sigma=\uparrow\downarrow} c^{\dagger}_{i,\sigma} c_{i,\sigma}] + ... \\ &= b - \gamma g \sum_{\sigma=\uparrow\downarrow} c^{\dagger}_{i,\sigma} c_{i,\sigma}\end{aligned}$$

and

$$\begin{aligned}\tilde{c}_{i,\sigma'} &= e^{S} c_{i,\sigma'} e^{-S} \\ &= c_{i,\sigma'} + [S, c_{i,\sigma'}] + \frac{1}{2!}[S,[S,c_{i,\sigma'}]] + \frac{1}{3!}[S,[S,[S,c_{i,\sigma'}]]] + ... \\ &= c_{i,\sigma'} + \gamma g \sum_{\sigma=\uparrow\downarrow} [c^{\dagger}_{i,\sigma} c_{i,\sigma}, c_{i,\sigma'}](b^{\dagger} - b) + \frac{1}{2!}[S,[S,c_{i,\sigma'}]] + \frac{1}{3!}[S,[S,[S,c_{i,\sigma'}]]] + ... \\ &= c_{i,\sigma'} - \gamma g (b^{\dagger} - b) c_{i,\sigma'} + \frac{1}{2!}(\gamma g)^2 (b^{\dagger} - b)^2 c_{i,\sigma'} - \frac{1}{3!}(\gamma g)^3 (b^{\dagger} - b)^3 c_{i,\sigma'} + ... \\ &= c_{i,\sigma'} \sum_{k=0}^{+\infty} (-1)^k \frac{(\gamma g)^k}{k!} (b^{\dagger} - b)^k \\ &= c_{i,\sigma'} e^{-\gamma g (b^{\dagger} - b)}.\end{aligned}$$

Conversely, for site $i \neq j$ we obtain:

$$\begin{aligned}\tilde{c}_{j,\sigma'} &= e^{S} c_{j,\sigma'} e^{-S} \\ &= c_{j,\sigma'} + [S, c_{j,\sigma'}] + \frac{1}{2!}[S,[S,c_{j,\sigma'}]] + \frac{1}{3!}[S,[S,[S,c_{j,\sigma'}]]] + ... \\ &= c_{j,\sigma'} + \gamma\, g \sum_{\sigma=\uparrow\downarrow} [c^{\dagger}_{i,\sigma} c_{i,\sigma}, c_{j,\sigma'}](b^{\dagger} - b) + \frac{1}{2!}[S,[S,c_{j,\sigma'}]] + \frac{1}{3!}[S,[S,[S,c_{j,\sigma'}]]] + ... \\ &= c_{j,\sigma'}\end{aligned}$$

In summary, the transformed operators are

$$\tilde{b} = b - \gamma g \sum_{\sigma=\uparrow\downarrow} c^{\dagger}_{i,\sigma} c_{i,\sigma} \qquad \tilde{b}^{\dagger} = b^{\dagger} - \gamma g \sum_{\sigma=\uparrow\downarrow} c^{\dagger}_{i,\sigma} c_{i,\sigma} \tag{S17}$$

$$\tilde{c}_{i,\sigma} = c_{i,\sigma} \Lambda \qquad \tilde{c}^{\dagger}_{i,\sigma} = c^{\dagger}_{i,\sigma} \Lambda^{\dagger} \tag{S18}$$

$$\tilde{c}_{j,\sigma} = c_{j,\sigma} \qquad \tilde{c}^{\dagger}_{j,\sigma} = c^{\dagger}_{j,\sigma}, \tag{S19}$$

where we have introduced $\Lambda = e^{-\gamma g(b^\dagger - b)}$. We now need to combine these operators to get the transformed Hamiltonian

$$\begin{aligned}\tilde{H}_{\chi,v} &= e^S H_{\chi,v} e^{-S} \\ &= \tilde{H}_\chi + \varepsilon \sum_\sigma \tilde{c}^\dagger_{i\sigma} \tilde{c}_{i\sigma} + \ \hbar\omega_0 \left(\tilde{b}^\dagger \tilde{b} + \frac{1}{2} \right) + g \left(\tilde{b} + \tilde{b}^\dagger \right) \tilde{n}_i.\end{aligned}$$

Rewriting term by term we obtain:

$$\tilde{n}_{i\sigma} = \tilde{c}^\dagger_{i,\sigma} \tilde{c}_{i,\sigma} = c^\dagger_{i,\sigma} \Lambda^\dagger c_{i,\sigma} \Lambda = c^\dagger_{i,\sigma} c_{i,\sigma}$$

$$\tilde{b}^\dagger + \tilde{b} = b^\dagger + b - 2\gamma \ g \sum_\sigma c^\dagger_{i,\sigma} c_{i,\sigma}$$

$$\begin{aligned}\tilde{b}^\dagger \tilde{b} &= \Big(b^\dagger - \gamma g \sum_\sigma c^\dagger_{i,\sigma} c_{i,\sigma} \Big) \Big(b - \gamma g \sum_\sigma c^\dagger_{i,\sigma} c_{i,\sigma} \Big) \\ &= b^\dagger b - \left(b^\dagger + b \right) \gamma g \sum_\sigma c^\dagger_{i,\sigma} c_{i,\sigma} + (\gamma g)^2 \Big[\sum_\sigma c^\dagger_{i,\sigma} c_{i,\sigma} \Big]^2.\end{aligned}$$

Therefore

$$\begin{aligned}\tilde{H}_{\chi,v} - H_\chi &= n_i \Big[\varepsilon + g \ (\tilde{b}^\dagger + \tilde{b}) \Big] + \hbar\omega_0 \Big(\tilde{b}^\dagger \tilde{b} + \frac{1}{2} \Big) \\ &= n_i \Big[\varepsilon + g(b^\dagger + b) - 2\gamma g^2 n_i \Big] + \hbar\omega_0 \Big[b^\dagger b + \frac{1}{2} - \gamma g \left(b^\dagger + b \right) n_i + \gamma^2 g^2 n_i^2 \Big] \\ &= \varepsilon \ n_i + \hbar\omega_0 \Big(b^\dagger b + \frac{1}{2} \Big) + n_i \left(b^\dagger + b \right) \Big(g - \gamma \ g\hbar\omega_0 \Big) - \gamma^2 \ \hbar\omega_0 \ g^2 n_i^2.\end{aligned}$$

From this we observe that by choosing $\gamma = 1/\hbar\omega_0$ the coupling is canceled and the transformed Hamiltonian becomes

$$\begin{aligned}\tilde{H}_{\chi,v} - H_\chi &= \varepsilon \ n_i + \hbar\omega_0 \Big(b^\dagger b + \frac{1}{2} \Big) - \frac{g^2}{\hbar\omega_0} n_i^2 \\ &= \left(\varepsilon - \frac{g^2}{\hbar\omega_0} \right) \ n_i + \hbar\omega_0 \Big(b^\dagger b + \frac{1}{2} \Big) - 2 \frac{g^2}{\hbar\omega_0} n_{i\uparrow} n_{i\downarrow},\end{aligned}$$

where we have exploited the relationship

$$n_i^2 = \Big[\sum_{\sigma=\uparrow\downarrow} c^\dagger_{i,\sigma} c_{i,\sigma} \Big]^2 = \Big[n_{i,\uparrow} + n_{i,\downarrow} \Big]^2 = n_{i,\uparrow} + n_{i,\downarrow} + 2 n_{i,\uparrow} n_{i,\downarrow}$$

to obtain the effective Hamiltonian

$$
\begin{aligned}
\tilde{H}_\chi = & \left(\varepsilon - \frac{g^2}{\hbar\omega_0}\right) n_i + \left(U - \frac{2g^2}{\hbar\omega_0}\right) n_{i\uparrow} n_{i\downarrow} \\
& + \left[-t\sum_\sigma c^\dagger_{i-1,\sigma} c_{i,\sigma} + i\lambda \sum_{\sigma\sigma'} c^\dagger_{i-2,\sigma} \boldsymbol{v}_{i-2}\cdot\boldsymbol{\sigma} c_{i,\sigma'}\right]\Lambda \\
& + \left[-t\sum_\sigma c^\dagger_{i,\sigma} c_{i+1,\sigma} + i\lambda \sum_{\sigma\sigma'} c^\dagger_{i,\sigma} \boldsymbol{v}_{i}\cdot\boldsymbol{\sigma} c_{i+2,\sigma'}\right]\Lambda^\dagger \\
& + U\sum_{j\neq i} n_{j\uparrow} n_{j\downarrow} - t \sum_{j\neq i,i-1}\sum_\sigma c^\dagger_{j,\sigma} c_{j+1,\sigma} + \\
& + i\lambda \sum_{j,\neq i,i-2}\sum_{\sigma\sigma'} c^\dagger_{j,\sigma} \boldsymbol{v}_j \cdot \boldsymbol{\sigma} c_{j+2,\sigma'} + \text{h.c.}\ ,
\end{aligned}
\tag{S20}
$$

with the purely bosonic term $\hbar\omega_0(b^\dagger b + 1/2)$ omitted. The re-normalized energy gap and Coulomb repulsion on site i are given on the first line of Eq. (S20). The reduction of the hopping and SOC terms involving site i is described by the the operator Λ on the second line. The remaining terms of the Hamiltonian (not involving site i) remain unaltered compared to Eq. (S14). Note that the transformation is *exact* (non perturbative) and it can be easily extended to a more general situation with different modes coupled to different sites.

From Eq. (S20) it is clear that an effective orbital degeneracy can be restored by properly choosing ε. Let us consider the situation sketched in Fig. 4-(a), with site 4 characterized by an energy gap $\varepsilon \gg \lambda$ and coupled to a vibrational mode. To understand how this coupling can amplify the effect of SOC, we need to consider the many-body states $|\psi_j^{N+1}\rangle$ with a double occupation either on 2 or 4. Due to the effect of vibrations both on one- and two-body terms in the first line of Eq. (S20), the energy of states with a double occupation on 4 is reduced by an amount $3g^2/\hbar\omega_0$. Hence, choosing $\varepsilon = 3g^2/\hbar\omega_0$ practically restores the degenerate situation of Fig. 1-(a), with the maximum effect of the SOC.
A few comments are in order. First, we note that this regime is perfectly realistic. Indeed, this condition is met by setting for instance $\varepsilon = 0.3$ eV and $\hbar\omega_0 = 0.1$ eV in the polaronic regime with $g \approx \hbar\omega_0$. Second, we do not need a perfect match between the energy gap and the bosonic renormalization to obtain a sizable polarization. This is demonstrated by simulations reported in Fig. 4 of the main text, where the degenerate situation is practically restored even using parameters which are not fine-tuned to exactly satisfy the aforementioned resonance condition. Finally, we note that, while simulations in the main text are performed

with the full Hamiltonian H_χ, the above analysis did not take into account the factor Λ which alters the effective hopping and SOC parameters in Eq. (S20) and hence changes the molecular spectrum.

VIII. RELAXATION

We describe thermal relaxation of the D$-\chi-$A supramolecule after ET by considering its interaction with a boson bath in the secular approximation [S14, S15], through modulation of the different terms of the Hamiltonian. Here we are especially interested in the long-time state after complete relaxation, much slower than the dynamics simulated in the main text. In particular, we perform simulations on a coarse-grained time scale, significantly longer than the inverse of the relevant energy gaps in the molecular spectrum, as required by the secular approximation to hold [S16]. Since relaxation is expected to occur on a much slower timescale than ET, we simulate it starting from the density matrix obtained after ET. To this end, we compute (in the low-temperature limit) the rate matrix accounting for the transition probability between the eigenstates of the whole supramolecule

$$W_{\mu\leftarrow\nu} = \begin{cases} \gamma_j |\langle\psi_\mu| H_j |\psi_\nu\rangle|^2 \Theta(E_\nu - E_\mu) & \text{for } \mu \neq \nu \\ -\sum_{\mu\neq\nu} W_{\mu\leftarrow\nu} & \text{for } \mu = \nu \end{cases} \tag{S21}$$

where E_μ is the energy of eigenstate $|\psi_\mu\rangle$ and the Heaviside function $\Theta(E_\nu - E_\mu)$ accounts for the bath spectral density and Bose-Einstein factor in the wide-band and low temperature limits. H_j are Hamiltonian terms modulated by the interaction between the system and vibrations. We consider, in particular, one-body terms such as on-site orbital energies H_0 and nearest-neighbors hopping H_t. Moreover, we include a weak isotropic exchange interaction between the electron sitting on the ground state of the donor and that on the first site of the bridge ($H_{D1} = \mathbf{S}_D \cdot \mathbf{S}_1$) and an analogous coupling between an electron on the last site of the chain and that on the acceptor ($H_{4A} = \mathbf{S}_4 \cdot \mathbf{S}_A$). A possible modulation of the spin-orbit coupling is also considered.

Then, we start from the density matrix obtained at the end of the ET process and we compute the time evolution of the diagonal elements of ρ by integrating

$$\dot{\rho}_{\mu\mu} = \sum_\nu W_{\mu\nu}\rho_{\nu\nu}. \tag{S22}$$

In the secular approximation, all coherences decay independently with rates $\frac{1}{2}(W_{\mu\mu} + W_{\nu\nu})$.

Results are reported in Fig. S5, where we have included a modulation of H_t, H_{D1} and H_{4A}. Since H_t is typically much stronger than H_{D1} and H_{4A}, we have assumed a factor of

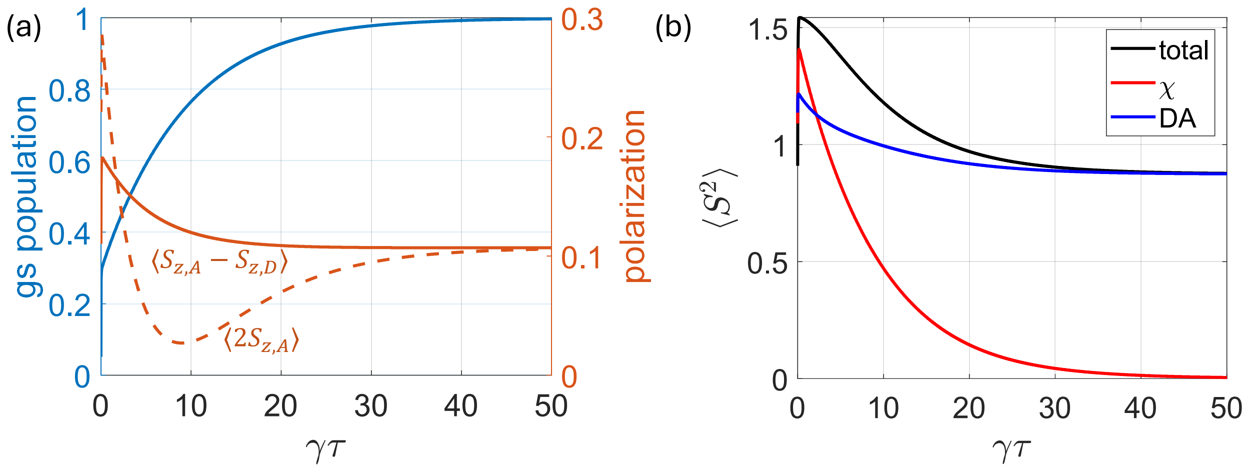

Figure S5: **System relaxation**. (a) Time evolution of the population of the bridge singlet ground state (left scale) and of the spin polarization (right scale) on the acceptor $2\langle S_{z,A}\rangle$ (dashed) or on the donor-acceptor pair $\langle S_{z,A} - S_{z,D}\rangle$ (solid), as defined in [S10, S17]. (b) Time evolution of the expectation vale of the total (black), bridge (red) or donor-acceptor spin. Time is in units of the slowest relaxation rate $\gamma \equiv \gamma_{D1} = \gamma_{4A}$.

100 also in their respective coupling with vibrations, yielding rates γ_j in a ratio of 10^4 in the rate matrix, Eq. (S21). The slowest time-scale allows for complete relaxation onto the ground singlet state of the bridge. Simultaneously, polarization initially on the bridge is distributed between D and A, due to the symmetric coupling assumed for the rates γ_{D1} and γ_{4A}. At the end, $\sim$ half of the original polarization survives on the acceptor and an opposite one arises on the donor.

Note that here for simplicity we have included only the υ_z component of the SOC but the picture is not changed by a more general choice. Inclusion of transverse terms of the SOC both in the Hamiltonian and in the coupling with the bath would reduce the (negative) polarization of the bridge, thus leading to a larger final net polarization on A. We have also checked that the inclusion of a modulation of H_0 or of the SOC does not significantly affect our results. In particular, we note that a modulation of the SOC is not needed to relax onto the bridge ground singlet, since S^2 (the square of the total spin of the D$-\chi-$A supra-molecule) is not a conserved quantity, even though all terms modulated by the bath are isotropic.

In Fig. S5-(b) we report the total spin of the system $S^2 = \left(\sum_{D,\chi,A} S_i\right)^2$, of the chiral

bridge $S_\chi^2 = \left(\sum_{i\in\chi} S_i\right)^2$ and of the DA pair $S_{DA}^2 = \left(\sum_{D,A} S_i\right)^2$. This highlights bridge relaxation to the ground state singlet, while $\langle S^2\rangle$ and $\langle S_{DA}^2\rangle$ converge to a value intermediate between that of a singlet (0) and of a triplet (2), as expected for a partially spin polarized state.

We finally note that to compare with experiments we have included a weak spin-spin dipolar coupling between the two unpaired electrons on D and A and an interaction with an external magnetic field of ~ 0.3 T, typical of X-band electron paramagnetic resonance experiments. This terms are very small compared to all other energy scales and hence they do not affect ET although they slightly modify the final D/A polarization after complete relaxation by changing the eigenstates of the DA radical pair. In Fig. S5 we used typical parameters of the system reported in [S11] [$\Delta g = 0.001$, dipole-dipole coupling of 4 MHz].

IX. LENGTH DEPENDENCE

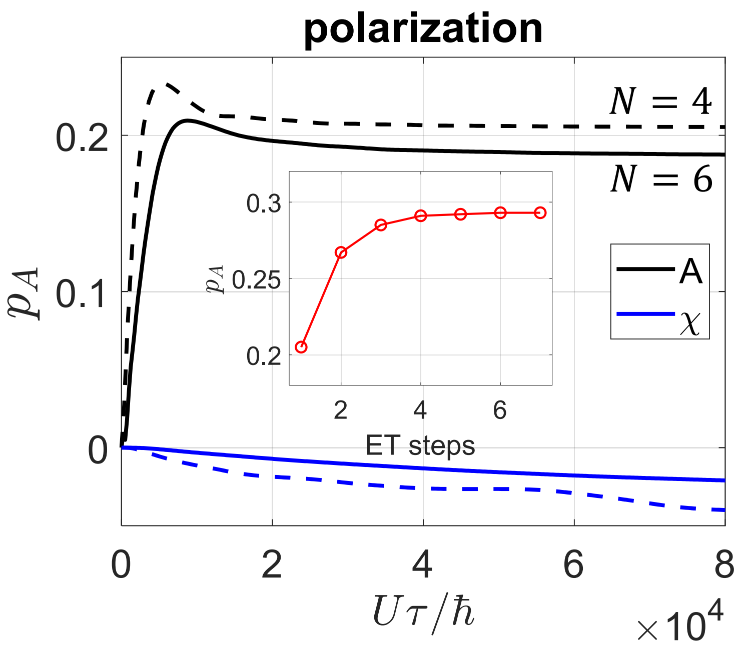

Figure S6: **Length dependence of the spin polarization**. Time evolution of the spin polarization on the acceptor site p_A (A, black) or on the chiral bridge χ (blue) for a chain of $N = 4$ (dashed lines, as in the simulations of the main text) or $N = 6$ sites (solid). Inset: spin polarization on the acceptor in a multi-step ET process, where at each step we initialize the donor state in the previous spin state of the acceptor. Parameters of the simulations: $t/U = 0.0125$, $\lambda/U = 6.25 \times 10^{-4}$, $\Gamma/U = 2.5 \times 10^{-4}$.

[S1] M. Geyer, R. Gutierrez, and G. Cuniberti, Effective Hamiltonian model for helically constrained quantum systems within adiabatic perturbation theory: Application to the chirality-induced spin selectivity (CISS) effect, J. Chem. Phys. **152**, 214105 (2020).

[S2] J. Fransson, Chirality-induced spin selectivity: The role of electron correlations, J. Phys. Chem. Lett. **10**, 7126 (2019).

[S3] J. Fransson, Vibrational origin of exchange splitting and "chiral-induced spin selectivity, Phys. Rev. B **102**, 235416 (2020).

[S4] J. Fransson, Charge redistribution and spin polarization driven by correlation induced electron exchange in chiral molecules, Nano Lett. **21**, 3026 (2021).

[S5] A.-M. Guo and Q.-f. Sun, Spin-selective transport of electrons in dna double helix, Phys. Rev. Lett. **108**, 218102 (2012).

[S6] A.-M. Guo and Q.-F. Sun, Spin-dependent electron transport in protein-like single-helical molecules, Proceedings of the National Academy of Sciences **111**, 11658 (2014).

[S7] M. Geyer, R. Gutierrez, V. Mujica, and G. Cuniberti, Chirality-induced spin selectivity in a coarse-grained tight-binding model for helicene, J. Phys. Chem. C **123**, 27230 (2019).

[S8] D. Tupkary, A. Dhar, M. Kulkarni, and A. Purkayastha, Fundamental limitations in lindblad descriptions of systems weakly coupled to baths, Phys. Rev. A **105**, 032208 (2022).

[S9] T. P. Fay, L. P. Lindoy, and D. E. Manolopoulos, Spin-selective electron transfer reactions of radical pairs: Beyond the Haberkorn master equation, The Journal of Chemical Physics **149**, 064107 (2018).

[S10] T. P. Fay and D. T. Limmer, Origin of chirality induced spin selectivity in photoinduced electron transfer, Nano Lett. **21**, 6696 (2021).

[S11] H. J. Eckvahl, N. A. Tcyrulnikov, A. Chiesa, J. M. Bradley, R. M. Young, S. Carretta, M. D. Krzyaniak, and M. R. Wasielewski, Direct observation of chirality-induced spin selectivity in electron donor–acceptor molecules, Science **382**, 197 (2023).

[S12] J. Fransson, *Non-Equilibrium Nano-Physics: A Many-Body Approach* (Springer Dordrecht, 2010).

[S13] S. Bravyi, D. P. DiVincenzo, and D. Loss, Schrieffer–wolff transformation for quantum many-body systems, Annals of Physics **326**, 2793 (2011).

[S14] H.-P. Breuer and F. Petruccione, *The Theory of Open Quantum Systems* (Oxford University Press, 2007).

[S15] V. May and O. Kühn, *Charge and Energy Transfer Dynamics in Molecular Systems* (John Wiley Sons, Ltd, 2011).

[S16] K. Blum, *Density Matrix, Theory and Applications* (Springer-Verlag Berlin Heidelberg, 2012).

[S17] T. P. Fay, Chirality-induced spin coherence in electron transfer reactions, J. Phys. Chem. Lett. **12**, 1407 (2021).
